# Supplementary material for: ECG Indices Poorly Predict Left Ventricular Hypertrophy and Are Applicable Only in Individuals with Low Cardiovascular Risk
Source: J Clin Med. 2020 May 6;9(5):1364. doi: 10.3390/jcm9051364 (PMC7290685; doi:10.3390/jcm9051364)
Supplement: Supplementary file 1 [file jcm-09-01364-s001.pdf]

Table S1. Results of the left ventricular mass index* (calculated by the formula LVM_{BSA}) multivariable linear regression analysis in the study population.

Variable	Model 1				Model 2			
	unstandardized B	95% C.I. for B	standardized B**	*p*	unstandardized B	95% C.I. for B	standardized B**	*p*
Risck SCORE, %	-0.495	-1.001-0.010	-0.121	0.056	-0.487	-1.007-0.034	-0.119	0.068
BPs, mmHg	0.076	-0.014-0.166	0.065	0.100	0.108	-0.010-0.226	0.093	0.073
BPd, mmHg	-0.072	-0.214-0.070	-0.036	0.321	-0.149	-0.338-0.041	-0.073	0.124
HR, bpm	-0.132	-0.256--0.008	-0.070	0.037	-0.135	-0.261--0.010	-0.072	0.035
Fasting glucose, mg/dL	0.097	0.023-0.171	0.095	0.011	0.071	-0.011-0.152	0.069	0.089
120 min glucose, mg/dL	0.013	-0.030-0.055	0.023	0.560				
HOMA-IR	0.239	-0.368-0.846	0.034	0.441	0.238	-0.371-0.847	0.033	0.444
hsCRP, mg/L	0.307	-0.089-0.702	0.051	0.130	0.291	-0.104-0.685	0.048	0.149
HbA1c, %	4.029	1.335-6.723	0.112	0.003	3.056	-0.042-6.153	0.085	0.054
NT-proBNP, pg/mL	0.020	0.012-0.029	0.173	<0.001	0.018	0.009-0.027	0.154	<0.001
hs-TnT, pg/mL	0.510	0.158-0.862	0.120	0.005	0.446	0.084-0.807	0.104	0.016
Fasting insulin, μUL/mL	-0.196	-0.453-0.061	-0.067	0.135	-0.186	-0.444-0.072	-0.064	0.159
LVEF BP, %	-0.157	-0.401-0.087	-0.043	0.208	-0.091	-0.339-0.158	-0.025	0.475
LAVI, ml/m^2	0.692	0.494-0.891	0.231	<0.001	0.677	0.475-0.880	0.226	<0.001
P wave time, ms	0.142	0.005-0.280	0.074	0.043	0.137	-0.001-0.276	0.071	0.052
QRS time, ms	0.369	0.203-0.534	0.162	<0.001	0.366	0.201-0.531	0.161	<0.001
Sokolow-Lyon index, mm	0.166	-0.038-0.369	0.056	0.112	0.173	-0.030-0.376	0.058	0.095
Lewis index, mm	0.330	0.145-0.514	0.131	<0.001	0.305	0.120-0.490	0.122	0.001
Cornell index, mm	0.931	0.666-1.196	0.238	<0.001	0.899	0.633-1.165	0.230	<0.001
WHR	-6.116	-28.145-15.913	-0.028	0.587	-9.614	-31.712-12.484	-0.044	0.394
% fat	-95.549	-131.385--59.713	-0.341	<0.001	-96.419	-132.198--60.640	-0.344	<0.001
Total fat mass, kg	-0.950	-1.345--0.555	-0.416	<0.001	-0.987	-1.383--0.592	-0.432	<0.001
Total lean mass, kg	0.559	0.278-0.840	0.282	<0.001	0.576	0.295-0.856	0.291	<0.001

Legs fat mass, kg	-1.322	-2.193--0.450	-0.174	0.003	-1.227	-2.114--0.339	-0.162	0.007
Android fat mass, kg	-5.193	-7.843--2.544	-0.302	<0.001	-5.700	-8.375--3.026	-0.331	<0.001
Gynoid fat mass, kg	-3.616	-5.596--1.636	-0.242	<0.001	-3.450	-5.453--1.448	-0.231	0.001
Visceral mass, kg	-0.645	-3.390-2.100	-0.029	0.645	-1.420	-4.223-1.382	-0.064	0.321
A/G fat mass ratio	-3.543	-13.471-6.385	-0.038	0.485	-5.636	-15.741-4.469	-0.061	0.275
G/T fat mass ratio	-4.351	-80.013-71.311	-0.006	0.910	10.305	-66.318-86.927	0.013	0.792
A/T fat mass ratio	-73.188	-176.644-30.267	-0.076	0.166	-85.284	-189.530-18.962	-0.089	0.109
Legs/T fat mass ratio	-3.150	-32.828-26.529	-0.011	0.835	3.095	-26.988-33.178	0.010	0.840

SCORE, Systematic Coronary Risk Estimation; BPs, systolic blood pressure; BPd, diastolic blood pressure; mmHg, millimeters of mercury; HR, heart rate; bpm, beats per minute; HOMA-IR, homeostasis model assessment of insulin resistance; CRP, C-reactive protein; HbA1c, hemoglobin A1c; NT-proBNP, N-terminal pro-brain natriuretic peptide; hs- TnT, high-sensitivity troponin T; LVEF BP, left ventricular ejection fraction biplane Simpson's method; LAVI, left atrial volume index; WHR, waist-hip ratio; A, android; G, gynoid; T, total; GFR, glomerular filtration rate Cockcroft-Gault Equation; BMI, body mass index; Model 1: adjusted for age, sex, GFR, BMI; Model 2: model 1 + additional adjustment for: history of hypertension, diabetes, atrial fibrillation, myocardial infarction, coronary heart disease, heart failure, peripheral artery disease, stroke and BP≥140 and/or≥90 mmHg;

*The left ventricular mass (LVM) index was calculated by the formula LVM/BSA;

**Standardized for independent and dependent variables.

Table S2. Results of the left ventricular mass index* (calculated by the formula LVM_{BSA}) multivariable linear regression analysis in the study population.

Variable	Model 3				Model 4			
	unstandardized B	95% C.I. for B	standardized B**	*p*	unstandardized B	95% C.I. for B	standardized B**	*p*
Risck SCORE, %	-0.790	-1.279--0.301	-0.192	0.002	-0.775	-1.273--0.276	-0.189	0.002
BPs, mmHg	0.115	0.023-0.206	0.098	0.014	-	-	-	-
BPd, mmHg	0.001	-0.144-0.145	0.000	0.994	-	-	-	-
HR, bpm	-0.126	-0.253-0.001	-0.067	0.052	-0.132	-0.260--0.004	-0.070	0.044
Fasting glucose, mg/dL	0.119	0.044-0.195	0.117	0.002	0.085	0.002-0.168	0.084	0.044
HOMA-IR	0.617	0.016-1.219	0.087	0.045	0.561	-0.043-1.165	0.079	0.069
hsCRP, mg/L	0.464	0.062-0.865	0.077	0.024	0.426	0.027-0.825	0.071	0.037
HbA1c, %	5.127	2.402-7.853	0.142	<0.001	3.981	0.840-7.121	0.110	0.013
NT-proBNP, pg/mL	0.020	0.011-0.029	0.174	<0.001	0.018	0.009-0.027	0.153	<0.001
hs-TnT, pg/mL	0.605	0.247-0.963	0.142	0.001	0.529	0.162-0.896	0.124	0.005
Fasting insulin, μUL/mL	0.098	-0.143-0.338	0.033	0.427	0.072	-0.172-0.315	0.025	0.564
LVEF BP, %	-0.181	-0.432-0.069	-0.049	0.157	-0.112	-0.365-0.142	-0.030	0.390
LAVI, ml/m²	0.733	0.531-0.936	0.245	<0.001	0.716	0.510-0.922	0.239	<0.001
P wave time, ms	0.191	0.051-0.331	0.099	0.008	0.173	0.032-0.313	0.089	0.016
QRS time, ms	0.388	0.219-0.558	0.171	<0.001	0.377	0.209-0.546	0.166	<0.001
Sokolow-Lyon index, mm	0.100	-0.108-0.308	0.034	0.348	0.117	-0.090-0.323	0.039	0.270
Lewis index, mm	0.429	0.245-0.613	0.171	<0.001	0.386	0.201-0.572	0.154	<0.001
Cornell index, mm	0.951	0.679-1.223	0.243	<0.001	0.902	0.630-1.174	0.231	<0.001
WHR	14.320	-7.145-35.786	0.066	0.191	8.156	-13.494-29.806	0.037	0.461
% fat	-8.220	-38.357-21.917	-0.029	0.593	-15.064	-45.380-15.252	-0.054	0.330
Total fat mass, kg	0.295	0.093-0.496	0.129	0.004	0.240	0.033-0.447	0.105	0.023

Total lean mass, kg	0.795	0.547-1.043	0.402	<0.001	0.778	0.530-1.026	0.393	<0.001
Legs fat mass, kg	0.602	-0.065-1.269	0.079	0.077	0.587	-0.080-1.254	0.077	0.085
Android fat mass, kg	2.162	0.635-3.690	0.126	0.006	1.689	0.106-3.273	0.098	0.037
Gynoid fat mass, kg	1.512	0.202-2.822	0.101	0.024	1.372	0.058-2.686	0.092	0.041
Visceral mass, kg	3.766	1.649-5.883	0.170	0.001	2.982	0.766-5.198	0.135	0.009
A/G fat mass ratio	9.721	0.737-18.705	0.106	0.034	6.651	-2.619-15.920	0.072	0.160
G/T fat mass ratio	-68.361	-142.449-5.727	-0.089	0.071	-44.101	-119.689-31.486	-0.057	0.253
A/T fat mass ratio	96.249	8.459-184.039	0.100	0.032	72.742	-16.880-162.364	0.076	0.112
Legs/T fat mass ratio	-32.640	-61.027--4.252	-0.109	0.025	-22.750	-51.837-6.337	-0.076	0.126

SCORE, Systematic Coronary Risk Estimation; BPs, systolic blood pressure; BPd, diastolic blood pressure; mmHg, millimeters of mercury; HR, heart rate; bpm, beats per minute; HOMA-IR, homeostasis model assessment of insulin resistance; CRP, C-reactive protein; HbA1c, hemoglobin A1c; NT-proBNP, N-terminal pro-brain natriuretic peptide; hs- TnT, high-sensitivity troponin T; LVEF BP, left ventricular ejection fraction biplane Simpson's method; LAVI, left atrial volume index; WHR, waist-hip ratio; A, android; G, gynoid; T, total; GFR, glomerular filtration rate Cockcroft-Gault Equation; Model 3: adjusted for age, sex, GFR; Model 4: model 1 + additional adjustment for: history of hypertension, diabetes, atrial fibrillation, myocardial infarction, coronary heart disease, heart failure, peripheral artery disease, stroke and BP≥140 and/or≥90 mmHg;

*The left ventricular mass (LVM) index was calculated by the formula LVM/BSA;

**Standardized for independent and dependent variables.

Table S3. Results of the left ventricular hypertrophy* (LVMI calculated by the formula LVM_{Height}) multivariable logistic regression analysis in the study population.

Variable	Model 1				Model 2			
	**OR unstandardized**	**95% C.I.**	**OR standardized**	***p***	**OR unstandardized**	**95% C.I.**	**OR standardized**	***p***
BPs, mmHg	1.011	0.994-1.028	1.209	0.225	-	-	-	-
BPd, mmHg	1.001	0.972-1.032	1.014	0.927	-	-	-	-
HR, bpm	0.999	0.973-1.025	0.988	0.935	1.000	0.973-1.028	0.999	0.993
LVEF BP, %	0.972	0.923-1.022	0.849	0.267	0.979	0.928-1.032	0.885	0.428
LAVI, ml/m²	1.051	1.012-1.092	1.421	0.010	1.055	1.013-1.099	1.455	0.010
P wave time, ms	1.017	0.988-1.047	1.201	0.254	1.016	0.985-1.047	1.181	0.321
QRS time, ms	1.057	1.018-1.097	1.678	0.004	1.052	1.013-1.093	1.612	0.008
Sokolow-Lyon index, mm	1.037	0.991-1.085	1.293	0.117	1.036	0.989-1.086	1.288	0.132
Cornell index, mm	1.081	1.022-1.144	1.520	0.007	1.069	1.009-1.133	1.431	0.024
Lewis index, mm	1.057	1.015-1.100	1.588	0.007	1.055	1.013-1.099	1.568	0.010
NT-proBNP, pg/mL	1.001	1.000-1.003	1.222	0.162	1.001	0.999-1.003	1.222	0.214
hs-TnT, pg/mL	1.055	0.989-1.126	1.304	0.105	1.055	0.987-1.128	1.302	0.118
Fasting glucose, mg/dL	1.006	0.994-1.019	1.116	0.333	1.004	0.989-1.019	1.068	0.626
120 min glucose, mg/dL	1.006	0.998-1.014	1.260	0.134	1.007	0.999-1.015	1.323	0.081
Fasting insulin, µUL/mL	0.989	0.950-1.030	0.925	0.590	0.992	0.951-1.035	0.946	0.714
120 Insulin, µUL/mL	1.002	0.998-1.007	1.168	0.260	1.003	0.999-1.008	1.217	0.169
HbA1c, %	1.812	1.140-2.878	1.397	0.012	1.907	1.056-3.443	1.437	0.032
HOMA -IR	0.976	0.861-1.105	0.948	0.701	0.977	0.856-1.115	0.950	0.725
hsCRP, mg/L	1.027	0.972-1.085	1.100	0.337	1.027	0.971-1.086	1.097	0.358
WHR	32.235	0.255-4073.566	1.398	0.160	28.128	0.177-4478.682	1.379	0.187
% fat	0.004	0.000-22.803	0.657	0.209	0.004	0.000-32.326	0.661	0.230
Total fat mass, kg	0.927	0.855-1.005	0.501	0.067	0.915	0.840-0.996	0.445	0.041
Total lean mass, kg	0.975	0.919-1.035	0.765	0.403	0.971	0.913-1.032	0.730	0.346
Android fat mass, kg	0.733	0.433-1.239	0.686	0.246	0.645	0.365-1.138	0.587	0.130
Gynoid fat mass, kg	0.724	0.478-1.098	0.638	0.129	0.750	0.487-1.154	0.670	0.191
Legs fat mass, kg	0.841	0.698-1.012	0.618	0.067	0.858	0.707-1.042	0.655	0.122
Visceral mass, kg	1.337	0.802-2.229	1.316	0.265	1.190	0.693-2.041	1.178	0.529
A/G fat mass ratio	1.663	0.213-13.020	1.122	0.628	1.159	0.131-10.214	1.034	0.895
G/T fat mass ratio	0.172	0.000-24045616	0.953	0.854	15.629	0.000-5744515610	1.078	0.785

A/T fat mass ratio	67.807	0.000-10755906882906	1.096	0.749	4.701	0.000-2390797527149	1.034	0.910
Legs/T fat mass ratio	0.018	0.000-34.800	0.756	0.299	0.089	0.000-247.279	0.845	0.550
Risk SCORE, %	0.897	0.783-1.027	0.995	0.115	0.869	0.751-1.006	0.993	0.060

BPs, systolic blood pressure; BPd, diastolic blood pressure; mmHg, millimeters of mercury; HR, heart rate; bpm, beats per minute; LVEF BP, left ventricular ejection fraction biplane Simpson's method; LAVI, left atrial volume index; NT-proBNP, N-terminal pro-brain natriuretic peptide; hs- TnT, high-sensitivity troponin T; HbA1c, hemoglobin A1c; HOMA-IR, homeostasis model assessment of insulin resistance; CRP, C-reactive protein; WHR, waist-hip ratio; A, android; G, gynoid; T, total; SCORE, Systematic Coronary Risk Estimation; GFR, glomerular filtration rate Cockcroft-Gault Equation; BMI, body mass index; Model 1: adjusted for age, sex, GFR, BMI; Model 2: model 1 + additional adjustment for: history of hypertension, diabetes, atrial fibrillation, myocardial infarction, coronary heart disease, heart failure, peripheral artery disease, stroke and BP≥140 and/or≥90 mmHg;

*The left ventricular mass (LVM) index was calculated by the formula LVM/Height $m^{2.7}$, and the LVH was defined as LVMI ≥50 $g/m^{2.7}$ for men and ≥47 $g/m^{2.7}$ for women;

**Standardized for independent variables.

Table S4. Results of the left ventricular hypertrophy* (LVMI calculated by the formula LVM_{Height}) multivariable logistic regression analysis in the study population.

	Model 3				Model 4			
**Variable**	**OR unstandardized**	**95% C.I.**	**OR standardized**	***p***	**OR unstandardized**	**95% C.I.**	**OR standardized**	***p***
BPs, mmHg	1.015	1.000-1.030	1.307	0.056	-	-	-	-
BPd, mmHg	1.018	0.991-1.045	1.202	0.192	-	-	-	-
HR, bpm	1.004	0.981-1.028	1.047	0.730	1.004	0.979-1.029	1.045	0.754
LVEF BP, %	0.970	0.926-1.016	0.842	0.202	0.979	0.933-1.028	0.888	0.399
LAVI, ml/m^2	1.057	1.020-1.096	1.475	0.002	1.057	1.017-1.099	1.477	0.005
P wave time, ms	1.029	1.003-1.056	1.368	0.028	1.024	0.996-1.052	1.286	0.096
QRS time, ms	1.060	1.026-1.096	1.727	0.001	1.054	1.019-1.091	1.635	0.002
Sokolow-Lyon index, mm	1.007	0.968-1.047	1.047	0.745	1.013	0.973-1.056	1.099	0.525
Cornell index, mm	1.078	1.025-1.132	1.493	0.003	1.062	1.008-1.119	1.381	0.025
Lewis index, mm	1.070	1.033-1.108	1.762	<0.001	1.065	1.026-1.104	1.691	0.001
NT-proBNP, pg/mL	1.002	0.999-1.004	1.365	0.143	1.002	0.999-1.005	1.418	0.158
hs-TnT, pg/mL	1.078	1.012-1.147	1.447	0.019	1.070	1.006-1.139	1.398	0.032
Fasting glucose, mg/dL	1.014	1.002-1.027	1.295	0.021	1.011	0.996-1.025	1.208	0.155
120 min glucose, mg/dL	1.011	1.004-1.019	1.563	0.002	1.012	1.004-1.019	1.582	0.002
Fasting insulin, µUL/mL	1.047	1.011-1.086	1.384	0.011	1.043	1.006-1.083	1.348	0.024
120 Insulin, µUL/mL	1.008	1.003-1.013	1.636	0.004	1.008	1.003-1.014	1.676	0.003
HbA1c, %	2.224	2.465-3.375	1.567	<0.001	2.243	1.294-3.888	1.575	0.004
HOMA -IR	1.164	1.034-1.309	1.390	0.012	1.140	1.009-1.288	1.328	0.036
hsCRP, mg/L	1.038	0.985-1.094	1.141	0.165	1.035	0.982-1.090	1.128	0.204
WHR	3944.557	79.794-194996.189	2.221	<0.001	1752.297	27.345-112288.770	2.054	<0.001
% fat	154683.203	189.763-126087973.589	2.458	<0.001	36918.723	32.785-41573561.273	2.207	0.003
Total fat mass, kg	1.127	1.079-1.177	2.971	<0.001	1.113	1.064-1.64-1.164	2.649	<0.001
Total lean mass, kg	1.083	1.035-1.134	2.335	0.001	1.071	1.021-1.123	2.066	0.005
Android fat mass, kg	2.375	1.735-3.251	2.856	<0.001	2.172	1.558-3.028	2.563	<0.001
Gynoid fat mass, kg	1.890	1.451-2.461	2.428	<0.001	1.828	1.390-2.406	2.319	<0.001
Legs fat mass, kg	1.276	1.126-1.445	1.965	<0.001	1.275	1.117-1.454	1.960	<0.001
Visceral mass, kg	3.081	2.075-4.576	2.899	<0.001	2.713	1.777-4.142	2.571	<0.001
A/G fat mass ratio	24.300	4.441-132.955	2.057	<0.001	13.645	2.229-83.535	1.805	0.005

G/T fat mass ratio	0.000	0.000-1.180	0.645	0.052	0.000	0.000-2560.723	0.776	0.288
A/T fat mass ratio	28041203241873500000	22999236919.749-341884855569649000000000000000	2.638	<0.001	103160343272948000	27485161.303-387192794938195000000000000	2.336	<0.001
Legs/T fat mass ratio	0.000	0.000-0.018	0.485	0.001	0.000	0.000-0.356	0.585	0.024
Risk SCORE, %	0.905	0.800-1.025	0.995	0.117	0.863	0.746-0.998	0.993	0.047

BPs, systolic blood pressure; BPd, diastolic blood pressure; mmHg, millimeters of mercury; HR, heart rate; bpm, beats per minute; LVEF BP, left ventricular ejection fraction biplane Simpson's method; LAVI, left atrial volume index; NT-proBNP, N-terminal pro-brain natriuretic peptide; hs- TnT, high-sensitivity troponin T; HbA1c, hemoglobin A1c; HOMA-IR, homeostasis model assessment of insulin resistance; CRP, C-reactive protein; WHR, waist-hip ratio; A, android; G, gynoid; T, total; SCORE, Systematic Coronary Risk Estimation; GFR, glomerular filtration rate Cockcroft-Gault Equation; Model 3: adjusted for age, sex, GFR; Model 4: model 1 + additional adjustment for: history of hypertension, diabetes, atrial fibrillation, myocardial infarction, coronary heart disease, heart failure, peripheral artery disease, stroke and BP≥140 and/or≥90 mmHg;

*The left ventricular mass (LVM) index was calculated by the formula LVM/Height m2.7, and the LVH was defined as LVMI ≥50 g/m2.7 for men and ≥47 g/m2.7 for women;

**Standardized for independent variables.

Table S5. Results of the left ventricular mass LVM index* (calculated by the formula LVMHeight) multivariable linear regression analysis in the study population.

Variable	Model 1				Model 2			
	unstandardized B	95% C.I. for B	standardized B**	*p*	unstandardized B	95% C.I. for B	standardized B**	*p*
Risck SCORE, %	-0.199	-0.431-0.033	-0.094	0.093	-0.205	-0.443-0.033	-0.097	0.093
BPs, mmHg	0.037	-0.005-0.078	0.061	0.083	-	-	-	-
BPd, mmHg	-0.028	-0.094-0.037	-0.027	0.396	-	-	-	-
HR, bpm	-0.047	-0.104-0.010	-0.049	0.104	-0.050	-0.107-0.008	-0.051	0.092
Fasting glucose, mg/dL	0.052	0.018-0.086	0.099	0.003	0.037	0.000-0.074	0.071	0.051
HOMA-IR	0.286	0.009-0.563	0.078	0.044	0.279	0.001-0.557	0.076	0.049
hsCRP, mg/L	0.133	-0.048-0.314	0.043	0.151	0.124	-0.056-0.305	0.040	0.177
HbA1c, %	1.985	0.752-3.218	0.107	0.002	1.397	-0.020-2.814	0.075	0.054
NT-proBNP, pg/mL	0.009	0.005-0.013	0.152	<0.001	0.008	0.004-0.012	0.134	<0.001
hs-TnT, pg/mL	0.251	0.090-0.412	0.114	0.002	0.214	0.049-0.379	0.098	0.011
Fasting insulin, µUL/mL	-0.084	-0.202-0.034	-0.056	0.162	-0.083	-0.201-0.035	-0.055	0.170
LVEF BP, %	-0.080	-0.192-0.032	-0.042	0.162	-0.048	-0.162-0.065	-0.025	0.406
LAVI, ml/m²	0.323	0.232-0.414	0.210	<0.001	0.317	0.224-0.409	0.206	<0.001
P wave time, ms	0.065	0.001-0.128	0.065	0.046	0.061	-0.002-0.124	0.061	0.060
QRS time, ms	0,146	0,070-0,222	0,125	<0.001	0,144	0,068-0,220	0,123	<0.001
Sokolow-Lyon index, mm	0,078	-0,015-0,172	0,051	0.100	0,082	-0.011-0.175	0,053	0.085
Lewis index, mm	0,151	0.066-0.235	0.117	<0.001	0.138	0.053-0.222	0.107	0.002
Cornell index, mm	0,425	0,303-0,546	0,211	<0.001	0,406	0,284-0,528	0,202	<0.001
WHR	-0,481	-10.580-9.617	-0,004	0,926	-2,433	-12,549-7,683	-0,022	0,638
% fat	-42,993	-59.429—26.556	-0.298	<0.001	-43.214	-59.601--26.827	-0.300	<0.001
Total fat mass, kg	-0.586	-0.764--0.407	-0.498	<0.001	-0.603	-0.782--0.425	-0.513	<0.001
Total lean mass, kg	0.065	-0.066-0.195	0.063	0.332	0.074	-0.056-0.204	0.073	0.263
Legs fat mass, kg	-0.905	-1.301--0.508	-0.232	<0.001	-0.848	-1.251--0.445	-0.217	<0.001
Android fat mass, kg	-2.860	-4.068--1.651	-0.323	<0.001	-3.147	-4.363--1.930	-0.356	<0.001
Gynoid fat mass, kg	-2.410	-3.307--1.513	-0.313	<0.001	-2.318	-3.225--1.411	-0.301	<0.001
Visceral mass, kg	-0.178	-1.436-1.080	-0.016	0.781	-0.589	-1.872-0.694	-0.052	0.369
A/G fat mass ratio	-1.164	-5.715-3.387	-0.025	0.616	-2.290	-6.915-2.335	-0.048	0.332
G/T fat mass ratio	-5.642	-40.317-29.033	-0.014	0.750	1.944	-33.119-37.007	0.005	0.914
A/T fat mass ratio	-32.464	-79.884-14.956	-0.066	0.180	-39.392	-87.092-8.308	-0.080	0.106
Legs/T fat mass ratio	-2.268	-15.869-11.334	-0.015	0.744	1.212	-12.554-14.978	0.008	0.863

SCORE, Systematic Coronary Risk Estimation; BPs, systolic blood pressure; BPd, diastolic blood pressure; mmHg, millimeters of mercury; HR, heart rate; bpm, beats per minute; HOMA-IR, homeostasis model assessment of insulin resistance; CRP, C-reactive protein; HbA1c, hemoglobin A1c; NT-proBNP, N-terminal pro-brain natriuretic peptide; hs- TnT, high-sensitivity troponin T; LVEF BP, left ventricular ejection fraction biplane Simpson's method; LAVI, left atrial volume index; WHR, waist-hip ratio; A, android; G, gynoid; T, total; GFR, glomerular filtration rate Cockcroft-Gault Equation; BMI, body mass index; Model 1: adjusted for age, sex, GFR, BMI; Model 2: model 1 + additional adjustment for: history of hypertension, diabetes, atrial fibrillation, myocardial infarction, coronary heart disease, heart failure, peripheral artery disease, stroke and BP≥140 and/or≥90 mmHg;

*The left ventricular mass (LVM) index was calculated by the formula LVM/Height $m^{2.7}$;

**Standardized for independent and dependent variables.

Table S6. Results of the left ventricular mass LVM index* (calculated by the formula LVM_{Heihgt}) multivariable linear regression analysis in the study population.

Variable	Model 3				Model 4			
	unstandardized B	95% C.I. for B	standardized B	*p*	unstandardized B	95% C.I. for B	standardized B	*p*
Risck SCORE, %	-0.536	-0.781-0.291	-0.254	<0.001	-0.547	-0.793-0.300	-0.259	<0.001
BPs, mmHg	0.078	0.032-0.124	0.130	0.001	-	-	-	-
BPd, mmHg	0.046	-0.027-0.119	0.044	0.215	-	-	-	-
HR, bpm	-0.041	-0.105-0.023	-0.042	0.212	-0.046	-0.110-0.018	-0.047	0.160
Fasting glucose, mg/dL	0.076	0.038-0.114	0.144	<0.001	0.053	0.011-0.094	0.101	0.013
HOMA-IR	0.678	0.378-0.978	0.185	<0.001	0.623	0.324-0.922	0.170	<0.001
hsCRP, mg/L	0.300	0.098-0.503	0.097	0.004	0.272	0.072-0.472	0.088	0.008
HbA1c, %	3.176	1.805-4.546	0.171	<0.001	2.409	0.838-3.980	0.130	0.003
NT-proBNP, pg/mL	0.009	0.005-0.014	0.155	<0.001	0.008	0.003-0.013	0.134	0.001
hs-TnT, pg/mL	0.353	0.173-0.534	0.161	<0.001	0.306	0.122-0.489	0.139	0.001
Fasting insulin, µUL/mL	0.201	0.081-0.322	0.134	0.001	0.175	0.054-0.296	0.117	0.005
LVEF Biplane, %	-0.105	-0.232-0.021	-0.055	0.104	-0.071	-0.198-0.057	-0.037	0.277
LAVI, ml/m^2	0.368	0.266-0.471	0.239	<0.001	0.361	0.258-0.464	0.234	<0.001
P wave time, ms	0.117	0.046-0.187	0.117	0.001	0.100	0.029-0.170	0.100	0.006
QRS time, ms	0.167	0.081-0.253	0.143	<0.001	0.156	0.072-0.241	0.134	<0.001
Sokolow-Lyon index, mm	0.011	-0.094-0.116	0.007	0.839	0.022	-0.082-0.126	0.015	0.675
Lewis index, mm	0.263	0.170-0.355	0.203	<0.001	0.230	0.138-0.323	0.178	<0.001
Cornell index, mm	0.446	0.307-0.584	0.221	<0.001	0.409	0.271-0.546	0.203	<0.001
WHR	20.229	9.473-30.984	0.180	<0.001	15.918	5.131-26.706	0.142	0.004
% fat	27.853	12.763-42.943	0.193	<0.001	23.345	8.244-38.445	0.162	0,003
Total fat mass, kg	0.405	0.307-0.502	0.344	<0.001	0.371	0.271-0.472	0.316	<0.001
Total lean mass, kg	0.432	0.307-0.556	0.424	<0.001	0.413	0.289-0.537	0.406	<0.001
Legs fat mass, kg	0.886	0.555-1.218	0.227	<0.001	0.876	0.547-1.204	0.224	<0.001
Android fat mass, kg	3.047	2.306-3.788	0.344	<0.001	2.775	2.008-3.542	0.313	<0.001
Gynoid fat mass, kg	2.131	1.487-2.776	0.277	<0.001	2.025	1.383-2.667	0.263	<0.001
Visceral mass, kg	4.204	3.174-5.235	0.369	<0.001	3.749	2.670-4.827	0.329	<0.001
A/G fat mass ratio	12.097	7.635-16.560	0.255	<0.001	10.120	5.533-14.707	0.213	<0.001
G/T fat mass ratio	-72.197	-109.340--35.055	-0.182	<0.001	-55.603	-93.287--17.919	-0.140	0.004
A/T fat mass ratio	130.243	86.838-173.649	0.264	<0.001	114.360	70.225-158.496	0.232	<0.001
Legs/T fat mass ratio	-33.100	-47.283--18.917	-0.215	<0.001	-26.200	-40.667--11.734	-0.171	<0.001

SCORE, Systematic Coronary Risk Estimation; BPs, systolic blood pressure; BPd, diastolic blood pressure; mmHg, millimeters of mercury; HR, heart rate; bpm, beats per minute; HOMA-IR, homeostasis model assessment of insulin resistance; CRP, C-reactive protein; HbA1c, hemoglobin A1c; NT-proBNP, N-terminal pro-brain natriuretic peptide; hs- TnT, high-sensitivity troponin T; LVEF BP, left ventricular ejection fraction biplane Simpson's method; LAVI, left atrial volume index; WHR, waist-hip ratio; A, android; G, gynoid; T, total; GFR, glomerular filtration rate Cockcroft-Gault Equation; Model 3: adjusted for age, sex, GFR; Model 4: model 1 + additional adjustment for: history of hypertension, diabetes, atrial fibrillation, myocardial infarction, coronary heart disease, heart failure, peripheral artery disease, stroke and BP≥140 and/or≥90 mmHg;
*The left ventricular mass (LVM) index was calculated by the formula LVM/Height $m^{2.7}$;
**Standardized for independent and dependent variables.
